# An Evaluation of Osseointegration Outcomes Around Trabecular Metal Implants in Human Maxillaries Reconstructed with Allograft and Platelet-Rich Fibrin

**DOI:** 10.3390/biomimetics10110789

**Published:** 2025-11-20

**Authors:** Sana Imani Oroumieh, Hana Shah, Andrew Nordlund, Luis Ignacio De Bellis Tulle, Bruno Martins de Souza, Anshumi Desai, Vasudev Vivekanand Nayak, Juan Carlos Carvajal Herrera, Lukasz Witek, Paulo G. Coelho

**Affiliations:** 1 Department of Biochemistry and Molecular Biology, University of Miami Miller School of Medicine, Miami, FL 33136, USA; 2 Florida International University Herbert Wertheim College of Medicine, Miami, FL 33199, USA; 3 University of Miami Miller School of Medicine, Miami, FL 33136, USA; 4 Prosthetics Department, Faculty of Dentistry, University of Chile, Región Metropolitana, Santiago 8380544, Chile; 5 Laboratory of Biomaterials, Military Engineering Institute, Rio de Janeiro 22290-270, Rio de Janeiro, Brazil; 6 Biomaterials and Regenerative Biology Division, NYU College of Dentistry, New York, NY 10010, USA; 7 DeWitt Daughtry Family Department of Surgery, Division of Plastic Surgery, University of Miami Miller School of Medicine, Miami, FL 33136, USA; 8 Dr. John T. Macdonald Foundation Biomedical Nanotechnology Institute (BioNIUM), University of Miami, Miami, FL 33136, USA; 9 Department of Biomedical Engineering, NYU Tandon School of Engineering, Brooklyn, NY 11201, USA; 10 Hansjörg Wyss Department of Plastic Surgery, NYU Grossman School of Medicine, New York, NY 10016, USA; 11 Department of Oral and Maxillofacial Surgery, NYU College of Dentistry, New York, NY 10010, USA; 12 Sylvester Comprehensive Cancer Center, University of Miami Miller School of Medicine, Miami, FL 33136, USA

**Keywords:** platelet-rich fibrin, guided bone regeneration, trabecular bone, tantalum

## Abstract

Trabecular Metal^TM^ (TM) dental implants comprise a tantalum (Ta)-based biomimetic open-cell structure designed to replicate the structural, functional, and physiological properties of cancellous bone. Yet, the current literature primarily focuses on the evaluation of osseointegration outcomes surrounding TM implants in uncompromised bone environments and/or brief periods of observation in pre-clinical models. In addition, the performance of TM implants in bony defect environments reconstructed with allogenic grafts and bioactive molecules, such as platelet-rich fibrin (PRF), has not been thoroughly investigated. This longitudinal, randomized clinical trial comprised patients presenting with completely edentulous maxillaries. Guided Bone Regeneration (GBR) was performed using a cortico-cancellous allograft/PRF agglomerate. After 26 weeks, bone biopsies were obtained, followed by the insertion of a TM implant, after which patients were allowed to heal for 52 weeks for assessment of osseointegration. Qualitatively, histomicrographs at 26 weeks confirmed the presence of newly formed bone extending from the periphery of defects and along the direct surface of the allograft. TM implant biopsies at 52 weeks demonstrated osseointegration with bone ongrowth and ingrowth at the interconnected, porous trabecular region. These histological characteristics were consistent across all patients. No metal debris was detected, and the TM implants maintained their porous structure throughout the study period. TM implants placed in PRF-augmented allograft-reconstructed maxillae fostered a conducive environment for osseointegration. By leveraging the open-cell Ta structure, robust new bone formation was achieved without signs of adverse tissue reactions.

## 1. Introduction

Reconstructive techniques play a crucial role in maxillofacial surgery for the restoration of damaged tissue as a result of various factors, including but not limited to trauma, infection, and congenital abnormalities [1]. Of note, satisfying the functional and aesthetic requirements of alveolar defects poses a significant challenge [2]. For example, the loss of bone volume as a consequence of partial or total tooth loss is a significant risk factor that greatly compromises the indication of maxillary and/or mandibular implant-supported prosthetic treatment [3]. Additionally, alterations or changes in bone density attributes caused by metabolic and/or hormonal imbalances can affect the osseointegration timeline of implants, ultimately delaying the prosthetic reconstruction process [4,5,6]. Therefore, the successful placement and functioning of implants in vivo relies on the presence of a robust bone foundation [1,7,8]. As a result, surgical techniques like Guided Bone Regeneration (GBR) have been developed with the goal of increasing the residual bone volume [9,10,11,12].

Autografts are considered the gold standard for GBR due to numerous advantages, including their biocompatibility and osseoconductivity [13]. However, their use is frequently constrained by donor site morbidity and the limited supply of donor tissue [14]. This has resulted in an increase in the utilization of allografts, xenografts, and alloplasts—viable alternatives for the reconstruction of the alveolar ridge [13,14]. Of note, allogenic bone grafts like Puros^®^ cortical particulate allograft have attracted considerable interest for their capacity to preserve crucial biological characteristics of native bone while reducing immunogenicity [10,15,16,17]. More importantly, previous studies have demonstrated that the potency of Puros^®^ cortical particulate allografts can be further augmented with the incorporation of bioactive molecules [18]. One such bioactive agent is platelet-rich fibrin—a dense scaffold composed of a fibrin matrix polymerized in a four-molecule structure, classified as pure platelet-rich plasma, leucocyte- and platelet-rich plasma, pure platelet-rich fibrin (P-PRF), and leucocyte- and platelet-rich fibrin (L-PRF, or simply PRF) [18,19].

PRF consists primarily of platelets and leukocytes. Within these cell populations, macrophages play a direct role in promoting osteogenesis via nuclear factor kappa B [18,19]. Additionally, macrophages help maintain local mesenchymal stromal or progenitor cell availability and facilitate the removal of apoptotic osteoblasts, thereby initiating a paracrine signaling loop [18,20]. Moreover, PRF provides a natural framework for essential growth factors such as transforming growth factor beta (TGF-β), vascular endothelial growth factor (VEGF), and insulin-like growth factor 1 (IGF-1), all of which facilitate improved angiogenesis and ossification [21]. In combination with allograft, this method has been shown in the literature to improve bone regeneration outcomes [22]. Moreover, PRF induces gingival fibroblast cell migration, which contributes to rapid healing [23], in addition to reducing patient discomfort, decreasing infection rates, and mitigating inadequate wound closure outcomes [24].

On another note, the field of dental implantology has also witnessed notable advancements, such as the development of Trabecular Metal^TM^ implants (TM, ZimVie Dental, Palm Beach Gardens, FL, USA) [25]. To elaborate, TM implants comprise a tantalum (Ta)-based biomimetic open-cell structure—designed to replicate the structural, functional and physiological properties of cancellous bone [26]. TM implants have shown improved surface roughness characteristics relative to widely utilized titanium (Ti)-based implants [27]. Furthermore, the TM design presents a porous structure (up to 80% porosity) with a nanotextured surface topography [28]. In addition, the trabecular structure of the implant body presents increased surface area for improved osseointegration outcomes through bone ingrowth [28,29]. From a biomechanical perspective, the TM design presents a low modulus of elasticity, which can reduce stress shielding when used in monoblock or monolithic applications [30]. Lastly, TM implants have demonstrated clinical relevance through structural integrity during implant placement and the ability to withstand physiological loading during clinical function [28,31].

The mechanical properties and demonstrated clinical safety of TM position this implant design as appropriate for use in low-bone-density environments or where non-porous implant alternatives are likely to yield suboptimal osseointegration outcomes. Moreover, as PRF has been shown to accelerate bone regeneration and increase the regenerated bone density, it is postulated that the combinatory therapeutic strategy of allogenic grafts with PRF will serve as a viable technique for GBR for subsequent placement of TM implants. Yet, the current literature primarily focuses on the evaluation of osseointegration outcomes surrounding TM implants in in vitro settings, or uncompromised/native bone environments [15,16,26,32]. Building on previous findings, the primary objective and novelty of this study were to establish an informed clinical benchmark and to evaluate osseointegration outcomes concerning the use of TM implants following maxillofacial reconstructions—specifically in human maxillaries reconstructed with particulate allografts augmented with PRF.

## 2. Materials and Methods

### 2.1. Clinical Trial Design, Eligibility Criteria and Ethical Considerations

This longitudinal, randomized clinical trial was approved by the Institutional Review Board (University of Chile approval # 18155-cyq-uch). The Consolidated Standards of Reporting Trials (CONSORT) guidelines served as the basis for both the design and reporting of this study. Additionally, all applicable regulations concerning clinical trials with human participants were followed, including the ICH E6 Good Clinical Practice guidelines, the Declaration of Helsinki, Food and Drug Administration (FDA) regulations (21 CFR 50 and 56), the Health and Human Services (HHS) regulations (45 CFR 46 Subparts A, B, C, and D), and the Health Insurance Portability and Accountability Act (HIPAA) of 1996. Prior to participation, all potentially eligible patients were fully informed about the study’s objectives, methodology, potential benefits, and risks, and were provided with a written explanation of the study protocol.

The inclusion criteria for this study were patients presenting with completely edentulous maxillaries with sufficient bone height (≥10 mm) to accommodate TM dental implants but insufficient width to support 3.7 or 4.7 mm diameter implants. Participants were required to have normal laboratory values for CBC, glycemia, coagulation time, and sedimentation rate, with no systemic pathologies contraindicating oral surgery. Eligible subjects included non-heavy smokers, non-diabetic individuals, or diabetic patients under medical control. The exclusion criteria consisted of abnormal laboratory values for CBC, glycemia, coagulation time, and sedimentation rate; the presence of systemic pathologies that contraindicated oral surgery; and individuals who were heavy smokers or had uncontrolled diabetes. All patients who met the inclusion criteria signed an informed consent form before any study-related procedures or treatments were initiated. Patients were not provided a monetary incentive and were not participants in any other ongoing clinical study.

### 2.2. Surgical Procedures

#### 2.2.1. Grafting Surgery

Antibiotic treatment therapy (875/125 mg of Ambilan^®^ Bid and 15 mg of meloxicam (Pharma Investi, Santiago, Chile)) was administered beginning 1 day before the surgery and maintained for 7 days (at 12 h and 24 h intervals, respectively). Pre-operative rinsing of the oral cavity was performed with a 0.2% chlorhexidine antiseptic solution, and perioral skin disinfection was performed with iodine solution. The surgical procedures were performed under local anesthesia and intravenous sedation, monitored by an anesthetist. Local infiltration anesthesia was used with mepivacaine chlorhydrate (Carboplyina 1:100,000, Dentsply Italia SrL, Rome, Italy). A supracrestal full-thickness flap was created with posterior bilateral releases to access the alveolar ridge. A surgical guide was used to create perforations into the marrow space to enhance graft vascularization. Four screws were placed to represent the volume to be augmented. Puros^®^ Cortico-Cancellous Particulate Allograft (0.25–1 mm, ZimVie Dental, Palm Beach Gardens, FL, USA) was mixed with PRF (synthesized using a pre-established protocol where blood sample was taken without anticoagulant in 10 mL tubes and immediately centrifuged at 3000 rpm for 10 min at ambient temperature [33,34]). The PRF/allograft agglomerate was mixed in a 1:1 ratio manually for 10 s and shaped to fit the surgical site. The flap was repositioned, a double-layer suture was applied with 3–0 Vicryl to close the site, and 1 g of paracetamol was administered every 8 h post-surgery for 7 days. Follow-ups at 3 and 7 days post-operatively included inspection of the surgical site for infection or inflammation.

#### 2.2.2. Implant Surgery

**Figure 1:**
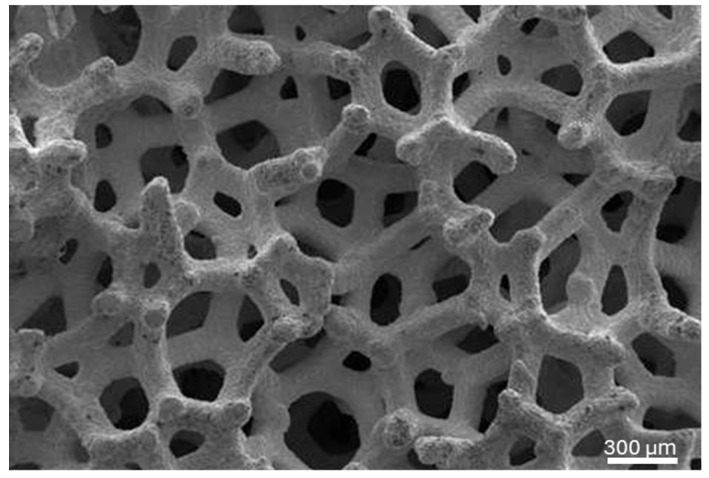
Representative scanning electron micrograph of the trabecular structure of the TM implants used in this study.

**Figure 2:**
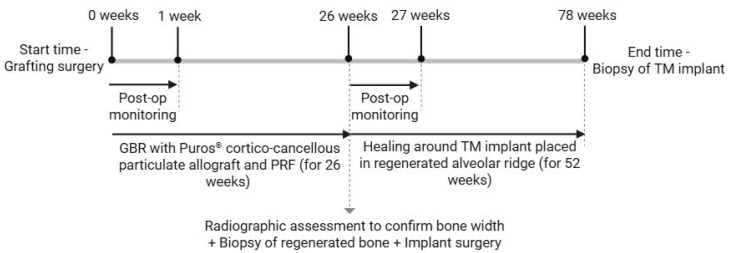
Timeline of the surgical interventions for the 78-week duration of the study.

Scanning electron microscopy was performed on the TM implants at 5 kV and working distance of 25 mm to visualize the trabecular structure of the implant body (Figure 1). Twenty-six weeks after grafting, a radiographic assessment was performed to assess sufficient bone width. Antibiotic treatment therapy (875/125 mg of Ambilan^®^ Bid and 15 mg of meloxicam (Pharma Investi, Santiago, Chile)) was administered beginning 1 day before the surgery and maintained for 7 days (at 12 h and 24 h intervals, respectively). During surgery, a supracrestal full-thickness flap was created, and a 4 mm diameter trephine bur was positioned perpendicular to the buccal aspect of the alveolar ridge at the level of the tooth to obtain a bone biopsy (per patient), followed by the insertion of a 3.7 mm × 10 mm TM implant (Trabecular Metal^TM^ Implant, ZimVie Dental, Palm Beach Gardens, FL, USA). Cover screws were placed, and the flap was sutured with 3–0 Vicryl to secure the implants during the healing period. 1 g of paracetamol was administered every 8 h post-surgery for 7 days. Follow-ups at 3 and 7 days post-operatively included inspection of the surgical site for infection or inflammation and suture removal, respectively. After healing for 52 weeks following implant placement (i.e., total time from initial grafting surgery = 78 weeks), TM implant biopsies were retrieved using a 4.3 mm trephine bur, and the sockets were filled with Puros^®^ Cortico-Cancellous Particulate Allograft (0.25–1 mm, ZimVie Dental, Palm Beach Gardens, FL, USA). A schematic timeline of the series of surgical interventions is provided in Figure 2.

### 2.3. Histological Preparation

En bloc samples (biopsies obtained at 26 and 52 weeks) were dehydrated using 70–100% ethanol (EtOH), subsequently immersed in methyl salicylate, and embedded in a methacrylate-based resin. Samples were sectioned along the longitudinal axis of the implant into ~300 μm thick slices using a low-speed precision wafering saw (Isomet 2000, Buehler Ltd., Lake Bluff, IL, USA). Slices were ground to a final thickness of 100 ± 5 μm with SiC abrasive papers (400, 600, 800, and 1200 grit) on a grinding machine (Metaserv 3000, Buehler, Lake Bluff, IL, USA) with abundant irrigation. Slides were polished with a micro-polish alumina-based solution (1 μm MicroPolish^TM^, Buehler, Lake Bluff, IL, USA) on a micro-fiber cloth for one minute.

### 2.4. Histomorphometric Analysis

Slides were stained with Stevenel’s Blue and Van Gieson picro-fuschin (SVG) and digitally scanned (Aperio CS2, Leica, Wetzlar, Germany) for quantitative histomorphometric analysis. For the 26-week bone samples (including the Puros^®^ Cortico-Cancellous Particulate Allograft/PRF mixture), the region of interest (ROI) was identified using the defect margins clearly visible on the histomicrographs to manually delineate bone, graft, and soft tissue by a single, trained investigator to ensure consistency between samples. %Bone, % Graft and %Soft Tissue (relative to the total area of the ROI) were quantified for each scan using customized computer software (JV Analysis, Biomaterials Division, New York University, New York, NY, USA; Department of Biochemistry and Molecular Biology, University of Miami, Coral Gables, FL, USA) [35]. For histomorphometric evaluation of the 52-week biopsies (that included the TM implant), ImageJ v 1.50i (National Institutes of Health, Bethesda, MD, USA) was used to quantify Bone-to-Implant Contact (BIC) and Bone Area Fractional Occupancy (BAFO) [36]. BIC was calculated as the percentage of the perimeter of newly formed bone in direct contact with the TM implant surface, whereas BAFO was calculated as the percentage of the bone area (ingrowth) within the trabecular (porous) portion of the TM implant macrogeometry.

## 3. Results

**Figure 3:**
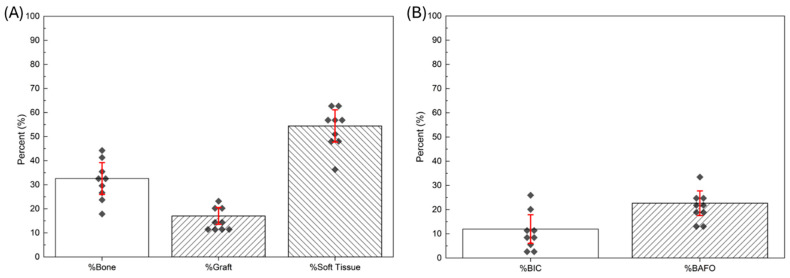
(**A**) %Bone, %Graft, and %Soft Tissue within the bone biopsies obtained after 26 weeks of healing following grafting surgery, and (**B**) %BIC and %BAFO within the trabecular region of the TM implants obtained after 52 weeks of healing following implant surgery. Scatter plots represent patient-level distributions.

Considering the inclusion/exclusion criteria, the clinical trial constituted enrollment of a total of *n* = 9 patients. No adverse events were reported among any of the patients included in the study. With respect to the bone biopsies retrieved 26 weeks following the initial grafting surgery for all patients, measurements of the outcome variables were averaged across all patients and presented as means and corresponding standard deviations (means ± SD) (Figure 3). New bone formed in the grafted region was 32.58% ± 6.6%, while remaining graft material and soft tissue were 17.04% ± 3.5% and 54.41% ± 6.7%, respectively. On the other hand, 52 weeks following implant surgery, %BIC and %BAFO values were 11.98% ± 5.9% and 22.64% ± 5.1%, respectively.

**Figure 4:**
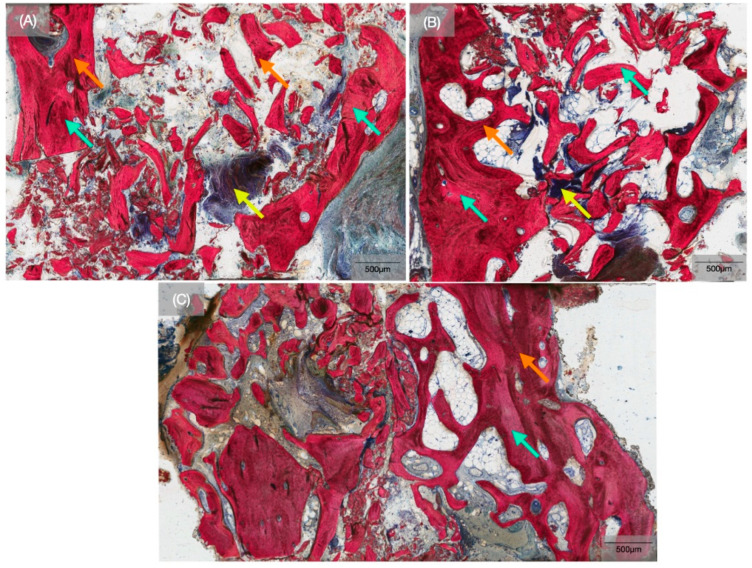
(**A**–**C**) Representative histomicrographs of the biopsies obtained from 3 different patients demonstrate the presence of allograft particles (teal arrows), bone marrow (yellow arrows), and new bone formation (orange arrow) at the 26-week time point.

**Figure 5:**
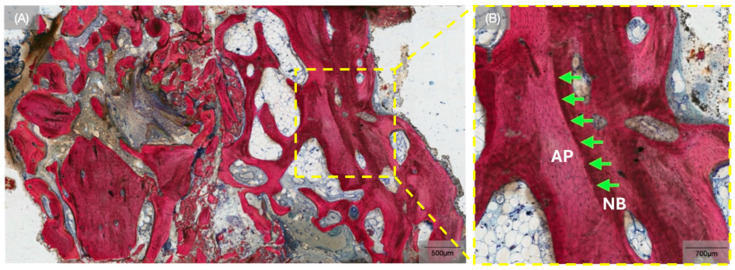
(**A**) Representative histomicrograph of the biopsy obtained at the 26-week time point, and (**B**) high magnification overview of the interface between newly forming bone (NB) and allograft particle (AP) (green arrows).

**Figure 6:**
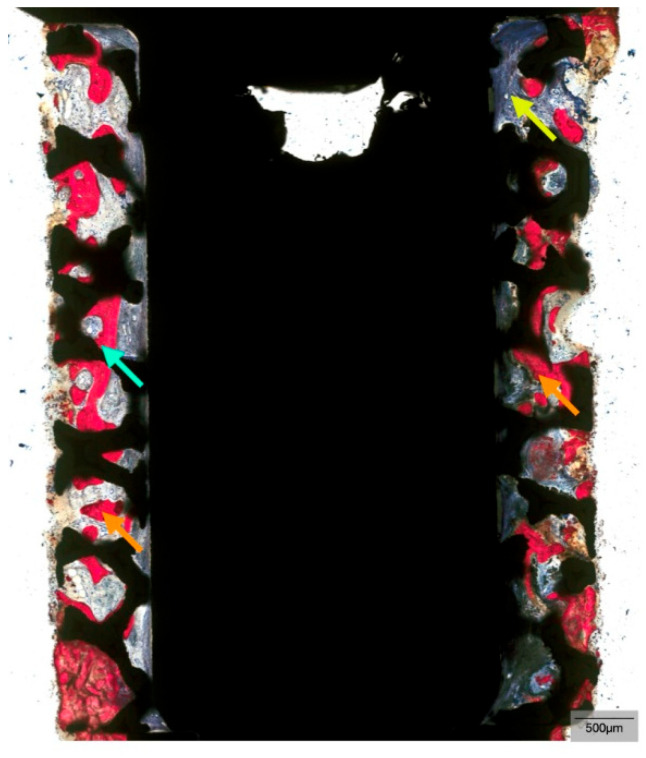
Representative histomicrograph of the TM implant demonstrating successful osseointegration along the entire length of the trabecular (porous) body of the TM implant after 52 weeks of healing. Direct bone-to-implant contact (teal arrow), bone marrow (yellow arrow), and new bone formation (orange arrows) can be noted.

Qualitatively, histomicrographs of the biopsies at the 26-week time point confirmed the presence of newly forming bone (Figure 4). SVG staining permitted the distinction between newly forming bone and allograft particles. The allograft particles were distinguishable by the clear boundaries separating them from newly forming bone and by their slight discoloration observed after staining (Figure 5). Newly forming bone could be seen extending from the periphery of defects and along the direct surface of the allograft (Figure 4). Considerable portions of the grafted area comprised fibrovascular connective tissue. Histomicrographs of the TM implant biopsies obtained after 52 weeks of healing demonstrated successful osseointegration with bone ongrowth and ingrowth at the interconnected, porous trabecular region of the implant, with similar histological features among all patients in the study (Figure 6). In addition, no metal debris was observed in the histomicrographs, demonstrating that the TM implants preserved the porous architecture for 52 weeks in vivo. Histological assessment facilitated the observation of a healing chamber-like effect created by the trabecular region of the implant macrogeometry. Additionally, woven bone formation within the porous healing chambers proceeded through an intramembranous-like healing pathway, wherein bone ingrowth originated from the osteotomy walls towards the trabecular region of the implant, as well as from the center of the healing chambers, with evidence of bone remodeling.

## 4. Discussion

The goal of alveolar ridge preservation (ARP) procedures is to mitigate post-extraction alveolar bone loss and ensure dimensional stability of the ridge during the healing process. For example, De Angelis et al. demonstrated the advantages of ARP compared to spontaneous healing in the reduction in horizontal and vertical ridge bone loss, with the former treatment modality improving healing outcomes and reducing the need for subsequent grafting procedures [2,37]. However, when this crucial clinical objective is not taken into account, GBR procedures of the residual alveolar ridge become essential to recover the lost bone volume and to ensure future placement of dental implants capable of efficiently supporting functional and aesthetic prosthetic rehabilitation. In addition, PRF’s role in the healing process is crucial as it serves as a reservoir for growth factors (i.e., VEGF, TGF-β, IGF-1) while promoting angiogenesis, stimulating osteoblast proliferation and differentiation, and facilitating osseointegration [28]. Moreover, PRF’s role in implant integration remains an area of active investigation. Previous studies have showcased its capacity to accelerate bone healing and increase implant stability after surgery, especially in cases involving compromised bone quality [38]. With new bone accounting for 32.58% ± 6.6% in the grafted sites at 26 weeks, our findings showcase the synergistic effect of PRF and graft material in supporting new bone formation. This aligns closely with the reported ranges of 30–40% seen in similar studies that utilize PRF and allogenic materials [39,40].

In combination with PRF, bone reconstruction has the potential to successfully enhance soft and hard tissue healing and regeneration [41]. The presence of soft tissue in the early healing phases reflects the normal progression of remodeling in grafted sites [42]. This aligns with our histological findings, which showed 54.41% ± 6.7% of the region contained soft tissue at 26 weeks. In a previously conducted long-term randomized clinical trial, similar outcomes were observed after 3 years with the use of bone allografts [43]. The study reported successful preservation with minimal resorption rates, providing further evidence for the utility of allografts in GBR [43]. Successful soft tissue integration, on the other hand, into the grafted region shows that structural integrity is maintained with minimal risk of ridge resorption—a common complication in cases of compromised maxillary bone [44,45].

On a separate note, qualitative evaluation of TM implants placed in grafted sites revealed positive outcomes of osseointegration after 52 weeks of healing, supporting their suitability following GBR. A limited number of studies have evaluated TM implants in settings of compromised bone conditions, where traditional Ti implants face challenges like bone resorption and poor tissue healing [25,46]. By mimicking cancellous bone, the trabecular design of the TM implant provides a porous scaffold-like bone environment that promotes osteoblast adhesion, vascular infiltration, and bone matrix deposition, creating a stable and biologically active interface [47]. Bobyn et al. first demonstrated that the highly porous nature of the Ta-based implants allowed for increased bone ingrowth and shear strength relative to Ti-based counterparts [48]. Furthermore, Fraser et al. demonstrated significantly higher BIC associated with Ta implants compared to Ti, with BIC values increasing from 34.6% ± 13.8% at 4 weeks to 50.8% ± 11.2% at 12 weeks [49]. This finding was supported by Al Deeb et al. and Lee et al., where a rapid and predictable level of osseointegration was observed between TM implants and native bone [27,47]. While these results are promising, it is important to note that animal models exhibit faster bone healing compared to humans [27]. Since our study focused on grafted human maxillary sites, we observed BIC and BAFO values of 11.98% ± 5.9% and 22.64% ± 5.1%, respectively, after 52 weeks of healing, congruent with the literature [25,26,50,51].

The results of this study underscore the clinical potential of combining TM implants with allogeneic grafts and PRF in reconstructive maxillofacial surgery. By combining advanced biomaterials with bioactive agents, the dual challenges of maintaining ridge dimensions and promoting robust bone formation can be addressed [52,53]. For example, El Chaar et al. demonstrated that TM implants have a 97.7% survival rate over a mean follow-up of 25 months, with minimal risk of failure in low-bone-density environments [54]. This supports the notion that TM implants can enhance secondary stability and be utilized in complex maxillary reconstructions, thereby improving prosthetic outcomes [25,26,50]. From a clinical perspective, this treatment modality would be beneficial in cases that require simultaneous implant placement and horizontal/vertical bone augmentation, simultaneous implant placement with sinus augmentation, or newly grafted sinuses/sockets. This multi-layered approach can also be used to enhance bone regeneration in compromised conditions and ensure long-term stability in prosthetic rehabilitation [25,45,47,51].

While the findings of this longitudinal study provide valuable insight into TM implant osseointegration in allograft-reconstructed bone, limitations exist. The single-institution setting limits the generalizability of these findings. Moreover, the small sample size, which can be attributed to the inclusion criteria, could also limit generalizability. Additionally, a lack of a randomized control group limited direct comparisons between TM implants and conventional Ti implants in the same grafted environment. Furthermore, extending follow-up periods beyond 52 weeks may further clarify the long-term stability of TM implants in grafted bone, as well as PRF’s extended effects on implant stability. For example, the survival rate of TM implants in a previous study was 100% after 1 year, which reduced to ~98% when TM implants were used in conjunction with extensive bone grafting and augmentation after a 2-year healing window [44]. In the same study, the TM group received a definitive prosthesis about 1–2 months earlier than traditional Ti implants [44]. As such, forthcoming studies evaluating long-term functional performance of TM implants could focus on further reducing implant failure rates in sites augmented with particulate bone graft and PRF relative to defect sites augmented with particulate bone graft alone, along with a robust computed tomographic and volumetric reconstructive analysis through the course of the healing period. Future studies addressing these limitations will ultimately guide clinicians toward more predictable and biologically informed strategies for complex maxillary reconstructions using TM implants placed in sites reconstructed with allogenic particulate grafts and PRF. Nonetheless, this study provides a foundation for future research aimed at optimizing biomaterial integration strategies to improve patient outcomes while highlighting the potential advantages of TM implants in dental implantology.

## Data Availability

The raw data supporting the conclusions of this article will be made available by the authors on request.
